# Effect of combination vaccines on completion and compliance of childhood vaccinations in the United States

**DOI:** 10.1080/21645515.2017.1362515

**Published:** 2017-09-07

**Authors:** Samantha K. Kurosky, Keith L. Davis, Girishanthy Krishnarajah

**Affiliations:** a RTI Health Solutions, Research Triangle Park, NC, USA; b GSK, Philadelphia, PA, USA

**Keywords:** adherence, immunization, infectious disease, pediatrics, United States, vaccines

## Abstract

Vaccination at age-appropriate intervals increases protection against morbidity and mortality; however, compliance rates among children remain low partly due to a complicated vaccination schedule. Use of combination vaccines reduces the number of injections per visit; however, there is limited evidence quantifying the effect of combination vaccines on vaccination rates. To examine how combination vaccines impact childhood completion (receipt of recommended doses) and compliance (receipt of age-appropriate vaccinations) rates, this study analyzed vaccination data from the 2012 National Immunization Survey (NIS), a nationally representative cross-sectional survey of caregivers of children aged 24 to 35 months in the United States. Vaccines were categorized as combination or single antigen. Vaccine completion was measured at ages 8, 18, and 24 months. Vaccine compliance and time undervaccinated were measured at 24 months. Children who received at least 1 combination vaccine (86%) had a higher completion rate (69%) and compliance with the full vaccine series (4:3:1:3:3:1:4 series) at 24 months (24%) than those who received only single-antigen vaccines (50% and 13%, respectively). Receipt of combination vaccine was associated with an increased likelihood of completing all recommended vaccinations at 24 months (odds ratio [OR] = 2.5; *P* < 0.001), receiving all vaccinations at age-appropriate times (OR = 2.2; *P* < 0.001), and less than 7 months undervaccinated (OR = 2.4; *P* < 0.001). Combination vaccines were associated with improved completion and compliance and should be encouraged among children who are undervaccinated or who received single-antigen vaccines only.

## Introduction

Despite the Advisory Committee on Immunization Practices' (ACIP's) recommendation that children receive numerous vaccinations by 2 y of age,^1^ compliance with the recommended vaccination schedule remains low.^2,3^ Evidence suggests the potentially large number of injections per visit and the complicated timing of each dose in the recommended childhood vaccination schedule can be cumbersome for parents and may lead to vaccination delay.^4-7^ For providers, developing a modified or catch-up schedule for children with delayed vaccination can be difficult due to the complicated timing of doses, thus resulting in missed opportunities for vaccination and further vaccination delay.^8^ Therefore, the ACIP suggests the use of combination vaccines (multiple antigens administered in the same syringe) as an effective strategy to simplify vaccination administration, thusly reducing barriers associated with simultaneous injections and the complicated dosing schedule.^9^

Several studies have quantified the effects of hepatitis B combination vaccines on the timely receipt of childhood vaccinations in the United States (US).^10,11^ A retrospective study of administrative Medicaid claims compared completion rates between children who received at least 1 dose of the diphtheria, tetanus toxoid and acellular pertussis adsorbed, hepatitis B (recombinant), and inactivated poliovirus (DTaP-HepB-IPV) combination vaccine and children who received no doses of combination vaccine.^10^ Results showed that children in the combination cohort had a higher completion rate for the recommended vaccination series as well as higher rates of individual antigens (i.e., DTaP, HepB, and IPV) compared with children in the reference cohort. Additionally, 45% of the combination cohort received all the recommended vaccinations at age-appropriate times, compared with 38% of the reference cohort. However, evidence related to the effect of other combination vaccines compared with single-antigen vaccines on completion and timeliness rates remains limited.

The objective of this study was to examine the effect of all currently available combination vaccines on completion of the recommended number of vaccine doses between birth and 2 y of age and to determine the age-appropriate receipt of each dose of childhood vaccination (compliance) among a nationally representative sample of children in the US.

## Results

A description of the study sample is presented in Table 1. The majority (86%) of children received at least 1 combination vaccine. Most of the mothers of the sampled children were aged 30 y or older (57%), married (63%), residing in the South (38%), and educated beyond high school (55%). About half of children were male, 53% were non-Hispanic white, and 75% had siblings in the household. Most children had 1 vaccine provider (67%), and most providers were based in private practice (58%). About half of children were enrolled in Medicaid or the SCHIP.

**Table 1.** Background child, family, and vaccination provider characteristics.

				Vaccine Type				
		Total		Received at Least 1 Combo Vaccine		No Combo Vaccines (Single-Antigen Only)		
Characteristic		n	%	n	%	n	%	*P* Value
Vaccine type	No combination vaccines	576 563	14.26	…	…	…	…	
	Had at least 1 combination vaccine	3 466 596	85.74	…	…	…	…	
Maternal age, years	< 20	70 396	1.74	61 283	1.77	9113	1.58	0.007
	20–30	1 674 987	41.43	1 474 115	42.52	200 873	34.84	
	> 30	2 297 776	56.83	1 931 198	55.71	366 577	63.58	
Maternal marital status	Married	2 534 567	62.69	2 140 025	61.73	394 542	68.43	0.015
Maternal education	< High school	749 284	18.53	647 610	18.68	101 674	17.63	< 0.001
	High school	1 082 417	26.77	945 624	27.28	136 793	23.73	
	> High school	943 370	23.33	834 419	24.07	108 951	18.90	
	College graduate	1 268 088	31.36	1 038 943	29.97	229 145	39.74	
Census region	Northeast	651 732	16.12	537 510	15.51	114 222	19.81	0.090
	South	1 552 917	38.41	1 351 164	38.98	201 754	34.99	
	Midwest	842 851	20.85	718 811	20.74	124 040	21.52	
	West	995 659	24.63	859 111	24.78	136 548	23.68	
Poverty status	At or below poverty line	1 458 429	36.07	1 271 180	36.67	187 249	32.48	0.007
	Above poverty line	2 400 123	59.36	2 025 724	58.44	374 399	64.94	
	Unknown	184 607	4.57	169 692	4.90	14 915	2.59	
Number of children in household	1	1 017 465	25.17	877 675	25.32	139 790	24.25	0.856
	2 or 3	2 428 384	60.06	2 081 045	60.03	347 340	60.24	
	4 or more	597 310	14.77	507 876	14.65	89 434	15.51	
Child's race/ethnicity	Non-Hispanic white	1 884 858	53.01	1 606 451	52.61	278 407	55.46	0.569
	Hispanic	1 113 340	27.54	957 999	27.64	155 341	26.94	
	Non-Hispanic black	557 206	13.78	488 968	14.11	68 238	11.84	
	Other	487 754	12.06	413 177	11.92	74 577	12.93	
Child's sex	Male	2 041 272	50.49	1 754 769	50.62	286 502	49.69	0.719
Number of vaccination providers for child	1	2 693 774	66.63	2 324 756	67.06	369 019	64.00	0.024
	2	1 099 518	27.19	920 049	26.54	179 469	31.13	
	3+	249 728	6.18	221 652	6.39	28 076	4.87	
Type of vaccination providers for child	All public	494 927	12.24	437 386	12.62	57 541	9.98	< 0.001
	All private	2 349 410	58.11	1 970 467	56.84	378 944	65.72	
	Other/mixed	1 198 822	29.65	1 058 743	30.54	140 078	24.30	
Had Medicaid or SCHIP	Yes	2 020 460	49.97	1 774 113	51.18	246 348	42.73	0.002

SCHIP = State Children's Health Insurance Program.

Demographic differences were observed between children who received at least 1 combination vaccine compared with those who received single-antigen vaccines only. Among children who received single-antigen vaccines only, a greater percentage of mothers were aged over 30 y (64% vs. 56%; *P* = 0.007), were married (68% vs. 62%; *P* = 0.015), graduated college (40% vs. 30%; *P* < 0.001), and had household incomes above the poverty line (65% vs. 58%; *P* = 0.007).

### Vaccine completion

For the majority of vaccines, children who received at least 1 combination vaccine had significantly (*P* < 0.001) higher completion rates compared with those who received single-antigen vaccines only (Table 2). Differences ranged from a low of a 16 percentage point difference for completion of pneumococcal conjugate vaccine (PCV) at 24 m to a high of a 20 percentage point difference for completion of IPV at 24 months. Completion of IPV, MMR, Hib, HepB, varicella, rotavirus, and PCV at 24 months was significantly lower among children who received single-antigen vaccines only compared with those who received combination vaccines. Completion of the full vaccine series (4 DTaP, 3 IPV, 1 MMR, 3 or 4 Hib, 3 HepB, 1 varicella, and 4 PCV) (4:3:1:3:3:1:4) was lower among those who received single-antigen vaccine only (50%) compared with children receiving combination vaccines (69%).

**Table 2.** Vaccine completion at 8, 18, and 24 months of age by receipt of combination vaccine.

			At 8 Months^a^^b^				At 18 Months^a^^c^				At 24 Months^a^^d^		
Completion Measure^a^		Total, %	Had at Least 1 Combo Vaccine, %	No Combo Vaccines (Single-Antigen Only), %	*P* Value^e^	Total, %	Had at Least 1 Combo Vaccine, %	No Combo Vaccines (Single-Antigen Only), %	*P* Value^e^	Total, %	Had at Least 1 Combo Vaccine, %	No Combo Vaccines (Single-Antigen Only), %	*P* Value^e^
DTaP	All	83.11	86.75	65.58	^*^	70.15	73.21	52.94	< 0.001	81.29	83.74	66.54	*
	None	3.44	0.00	20.01		2.16	0.00	14.32		1.75	0.00	12.24	
IPV	All	92.61	96.90	71.98	< 0.001	91.48	95.50	68.90	< 0.001	93.16	96.04	75.84	< 0.001
	None	3.79	0.01	22.02		2.72	0.02	17.88		2.37	0.02	16.48	
MMR	All	…	…	…		87.18	90.03	71.17	< 0.001	91.30	93.69	76.96	< 0.001
	None	…	…	…		12.82	9.97	28.83		8.70	6.31	23.04	
Hib (3- or 4-dose series)	All	81.30	84.79	64.49	< 0.001	72.94	75.73	57.27	< 0.001	80.13	82.39	66.58	< 0.001
	None	3.70	0.14	20.86		2.46	0.05	15.99		1.91	0.05	13.09	
Hib (4-dose series)	All	82.43	84.27	72.16	< 0.001	73.69	75.16	63.94	^*^	81.24	82.06	75.41	^*^
	None	1.63	0.10	10.13		0.49	0.00	3.71		0.00	0.00	0.00	
Hib (3-dose series)	All	91.69	96.51	75.67	< 0.001	87.48	88.68	82.82	^*^	90.48	90.14	91.97	^*^
	None	5.61	0.17	23.73		1.96	0.00	9.53		0.00	0.00	0.00	
HepB	All	91.51	95.76	71.02	< 0.001	88.27	91.74	68.81	< 0.001	89.77	92.59	72.84	< 0.001
	None	2.96	0.74	13.66		2.24	0.72	10.81		2.07	0.82	9.60	
Varicella	All	…	…	…		86.75	89.76	69.83	< 0.001	90.75	93.26	75.64	< 0.001
	None	…	…	…		13.25	10.24	30.17		9.25	6.74	24.36	
Rotavirus (2- or 3-dose series)	All	67.70	71.83	47.79	< 0.001	68.82	72.14	50.16	< 0.001	68.83	71.56	52.46	< 0.001
	None	16.61	12.02	38.70		16.56	12.75	37.95		16.56	13.41	35.51	
Rotavirus (3-dose series)	All	79.91	80.19	78.04	0.138	81.42	81.43	81.28	^*^	81.44	81.38	81.85	^*^
	None	0.05	0.03	0.20		0.00	0.00	0.00		0.00	0.00	0.00	
Rotavirus (2-dose series)	All	89.33	90.70	75.39	< 0.001	89.58	90.74	76.44	^*^	89.59	90.72	76.52	^*^
	None	0.06	0.04	0.29		0.00	0.00	0.00		0.00	0.00	0.00	
PCV	All	77.46	81.09	60.00	< 0.001	76.02	78.91	59.81	< 0.001	80.64	82.89	67.10	< 0.001
	None	4.95	1.19	23.08		3.33	0.79	17.63		2.70	0.76	14.38	
4:3:1:3:3:1	All	…	…	…		…	…	…		70.63	73.52	53.22	^*^
	None	…	…	…		…	…	…		0.28	0.00	1.93	
4:3:1:3:3:1:4	All	…	…	…		…	…	…		66.53	69.31	49.86	^*^
	None	…	…	…		…	…	…		0.24	0.00	1.69	

DTaP = diphtheria, tetanus toxoid, and acellular pertussis; HepB = hepatitis B; Hib = *Haemophilus influenzae* type b; IPV = inactivated poliovirus vaccine; MMR = measles, mumps, and rubella vaccine; PCV = pneumococcal conjugate vaccine.

a All percentage values are weighted based on National Immunization Survey sampling design. “All” is defined as having received all recommended doses by the measurement period. ”None” is defined as having received zero doses by the measurement period. Partial completion (having received at least 1 (but not all) doses by the measurement period) was a third category included in this analysis and can be calculated by subtracting the sum of “All” and “None” from 100%.

b Total count of doses received at 8 months includes 3 DTaP, 2 IPV, 1 or 2 Hib, 2 or 3 rotavirus, and 3 PCV.

c Total count of doses received at 18 months includes 4 DTaP, 3 IPV, 1 MMR, 3 or 4 Hib, 3 HepB, 1 varicella, 2 or 3 rotavirus, and 4 PCV.

d Total count of doses received at 24 months includes 4 DTaP, 3 IPV, 1 MMR, 3 or 4 Hib, 3 HepB, 1 varicella, 2 or 3 rotavirus, and 4 PCV.

e Tested on entire sample, including children who had partial completion.

* Chi-square statistics not calculated due to zero count cells.

### Vaccine compliance

Compliance rates for children who received combination vaccines was significantly (*P* < 0.001) higher from those who received single-antigen vaccines only (Table 3). For all vaccines, the proportion of children who received all doses on time was higher among those receiving combination vaccines compared with those who received single-antigen vaccines only. For example, 58% of those receiving combination vaccines received 4 doses of DTaP on time compared with 45% of those who received single-antigen vaccines only (*P* < 0.001). The largest statistically significant differences were observed for IPV (22%), HepB (22%), and varicella (19%). Children receiving combination vaccines had a higher compliance rate for the 4:3:1:3:3:1:4 series compared with those receiving single-antigen vaccines only (24% vs. 13%; *P* < 0.001). For most vaccines, the mean number of days undervaccinated among children who received combination vaccines was significantly lower than among those who received single-antigen vaccines only.

**Table 3.** Vaccine compliance: doses received by 24 months.

		Compliance Measure^a^										
					Vaccinated Late		Severe Undervaccination^b^		Total Number of Days Undervaccinated^c^			
Compliance Measure		No Doses on Time, %	All Doses on Time, %	*P* Value	%	*P* Value	%	*P* Value	Mean	SE	Median	*P* Value
4 DTaP	Total	7.94	56.47		43.53		13.92		204.32	5.67	150.24	
	At least 1 combo vaccine	5.59	58.34	< 0.001	41.66	< 0.001	11.54	< 0.001	178.34	5.21	150.04	< 0.001
	No combo vaccines	22.03	45.17		54.83		28.19		322.96	17.54	186.69	
3 IPV	Total	5.56	76.92		23.08		7.60		204.32	5.67	80.32	
	At least 1 combo vaccine	2.69	80.06	< 0.001	19.94	< 0.001	4.68	< 0.001	144.06	7.93	62.68	< 0.001
	No combo vaccines	22.83	58.05		41.95		25.17		364.92	18.18	399.16	
1 MMR	Total	21.14	78.86		21.14		9.80		147.59	3.48	168.39	
	At least 1 combo vaccine	18.56	81.44	< 0.001	18.56	< 0.001	7.37	< 0.001	135.27	3.91	116.96	< 0.001
	No combo vaccines	36.65	63.35		36.65		24.36		185.09	7.44	236.52	
Hib	Total	10.19	41.86		58.14		27.17		221.37	4.72	157.71	
	At least 1 combo vaccine	7.62	43.38	< 0.001	56.62	< 0.001	24.83	< 0.001	202.73	4.73	142.96	< 0.001
	No combo vaccines	25.65	32.75		67.25		41.25		315.73	15.24	241.70	
4 Hib	Total	8.13	43.25		56.75		24.90		203.20	4.66	130.97	
	At least 1 combo vaccine	7.32	44.04	< 0.001	55.96	< 0.001	23.80	0.113	197.76	4.95	126.35	< 0.001
	No combo vaccines	13.93	37.66		62.34		32.70		237.93	14.08	198.45	
3 Hib	Total	14.33	31.51		68.49		42.27		271.83	11.01	245.38	
	At least 1 combo vaccine	12.78	30.03	< 0.001	69.97	< 0.001	45.42	0.569	278.99	11.85	246.16	< 0.001
	No combo vaccines	21.12	37.98		62.02		28.48		236.51	28.01	162.77	
3 HepB	Total	7.48	61.34		38.66		11.94		207.34	6.79	106.65	
	At least 1 combo vaccine	5.96	64.45	< 0.001	35.55	< 0.001	8.95	< 0.001	171.88	6.48	99.26	< 0.001
	No combo vaccines	16.62	42.62		57.38		29.92		339.40	19.64	197.36	
1 varicella	Total	21.54	78.46		21.54		10.27		150.09	3.44	169.02	
	At least 1 combo vaccine	18.78	81.22	< 0.001	18.78	< 0.001	7.70	< 0.001	137.55	3.78	117.25	< 0.001
	No combo vaccines	38.14	61.86		38.14		25.72		187.23	7.15	241.23	
Rotavirus	Total	21.47	59.09		40.91		31.90		470.56	7.02	576.80	
	At least 1 combo vaccine	18.24	61.73	< 0.001	38.27	< 0.001	29.05	< 0.001	454.36	7.93	576.34	< 0.001
	No combo vaccines	40.85	43.20		56.80		49.06		536.17	13.99	637.21	
3 rotavirus	Total	5.71	69.94		30.06		19.52		364.66	10.32	504.73	
	At least 1 combo vaccine	5.44	70.22	< 0.001	29.78	0.445	19.36	0.197	364.21	10.95	504.60	< 0.001
	No combo vaccines	7.75	67.78		32.21		20.75		367.85	30.15	468.21	
2 rotavirus	Total	7.05	76.66		23.34		10.81		287.91	25.94	74.11	
	At least 1 combo vaccine	6.50	78.16	< 0.001	21.84	< 0.001	9.71	0.507	277.29	27.53	72.98	< 0.001
	No combo vaccines	13.46	59.37		40.63		23.48		354.10	79.02	200.66	
4 PCV	Total	11.35	50.47		49.53		50.48		250.73	5.99	189.79	
	At least 1 combo vaccine	8.63	52.47	< 0.001	47.53	< 0.001	47.53	< 0.001	229.78	6.34	152.61	< 0.001
	No combo vaccines	27.68	38.44		61.56		64.19		348.03	16.22	292.05	
4:3:1:3:3:1^d^	Total	3.06	25.86		74.14		38.25		254.44	4.52	205.70	
	At least 1 combo vaccine	0.93	27.56	< 0.001	72.44	< 0.001	35.37	< 0.001	233.09	4.45	174.63	< 0.001
	No combo vaccines	15.86	15.66		84.34		55.53		364.72	14.93	322.06	
4:3:1:3:3:1:4^e^	Total	3.06	22.89		77.11		42.65		276.06	4.64	241.13	
	At least 1 combo vaccine	0.93	24.46	< 0.001	75.54	< 0.001	39.85	< 0.001	255.61	4.72	220.88	< 0.001
	No combo vaccines	15.86	13.43		86.57		59.48		383.36	14.72	349.37	

DTaP = diphtheria, tetanus toxoid, and acellular pertussis vaccine; HepB = hepatitis B vaccine; Hib = *Haemophilus influenzae* type b vaccine; IPV = inactivated poliovirus vaccine; MMR = measles, mumps, and rubella vaccine; PCV = pneumococcal conjugate vaccine; SE = standard error of the mean.

a Compliance is an assessment of receipt of doses at age-appropriate intervals.

b Severe undervaccination is defined as having 7 months or more of cumulative undervaccination.

c Total number of days undervaccinated is the total count of cumulative days undervaccinated.

d The 4:3:1:3:3:1 series includes 4 DTaP, 3 IPV, 1 MMR, 3 or 4 Hib, 3 hepatitis B, and 1 varicella recommended by age 24 months.

e The 4:3:1:3:3:1:4 series includes 4 DTaP, 3 IPV, 1 MMR, 3 or 4 Hib, 3 hepatitis B, 1 varicella, and 4 PCV recommended by age 24 months.

### Likelihood of completion and compliance

Findings from multivariable logistic regression on 3 completion and compliance outcomes (i.e., completed series by 24 months, received all vaccines on time, and less than 7 months of undervaccination) indicated receiving combination vaccines was significantly associated with improved outcomes after controlling for individual, household, and provider characteristics (Table 4). For the 4:3:1:3:3:1:4 series, the likelihood of completing all vaccines in the series by 24 months was 2.5 times greater for children receiving combination vaccines compared with those receiving single-antigen vaccines only (*P* < 0.001). Similarly, compared with children receiving single-antigen vaccines only those receiving at least 1 combination vaccine were 2.2 times more likely to receive all vaccines on time and 2.4 times more likely to have less time undervaccinated (less than 7 months).

**Table 4.** Factors associated with completion and compliance measured at 24 months.

		Completed Series by 24 Months^a^				All Vaccines on Time^a^				Time Undervaccinated (< 7 Months)^a^			
Completion and Compliance by Series		Odds Ratio	95% LCL	95% UCL	*P* Value	Odds Ratio	95% LCL	95% UCL	*P* Value	Odds Ratio	95% LCL	95% UCL	*P* Value
4:3:1:3:3:1													
Vaccine type^b^	Had at least 1 combination vaccine	2.668	2.162	3.293	< 0.001	2.113	1.702	2.623	<0.001	2.437	1.984	2.995	< 0.001
Maternal age, years^c^	20–30	0.855	0.497	1.471	0.412	0.730	0.405	1.318	0.335	0.792	0.467	1.342	0.383
	> 30	0.935	0.540	1.619	0.944	0.729	0.401	1.325	0.342	0.808	0.474	1.378	0.522
Maternal marital status^d^	Married	0.821	0.679	0.992	0.041	0.835	0.688	1.013	0.067	0.912	0.760	1.095	0.324
Maternal education^e^	High school	1.238	0.962	1.594	0.377	1.065	0.826	1.374	0.229	1.155	0.903	1.478	0.088
	> High school	1.375	1.068	1.771	0.533	1.278	0.968	1.687	0.219	1.391	1.091	1.774	0.301
	College graduate	1.774	1.339	2.352	< 0.001	1.372	1.030	1.829	0.035	1.785	1.366	2.332	< 0.001
Poverty status^f^	Below poverty line	1.106	0.892	1.372	0.223	0.981	0.779	1.236	0.524	1.024	0.834	1.257	0.396
	Unknown	0.903	0.622	1.312	0.407	1.131	0.793	1.614	0.446	0.856	0.600	1.221	0.339
Number of vaccination providers for child^g^	2	0.899	0.747	1.082	0.836	0.831	0.683	1.010	0.567	0.776	0.647	0.930	0.277
	3+	0.771	0.543	1.095	0.234	0.785	0.577	1.068	0.331	0.759	0.557	1.035	0.336
Type of vaccination providers for child^h^	Other/mixed	1.088	0.815	1.452	0.700	1.016	0.744	1.389	0.423	1.256	0.955	1.653	0.166
	Private	1.279	0.972	1.683	0.027	0.870	0.650	1.163	0.119	1.213	0.932	1.579	0.375
Had Medicaid or SCHIP^i^	Yes	0.964	0.793	1.173	0.716	0.833	0.675	1.028	0.089	0.930	0.770	1.124	0.454
4:3:1:3:3:1:4													
Vaccine type^b^	Had at least 1 combination vaccine	2.501	2.029	3.082	< 0.001	2.177	1.732	2.736	< 0.001	2.398	1.947	2.953	< 0.001
Maternal age, years^c^	20–30	0.775	0.457	1.316	0.235	0.675	0.372	1.227	0.206	0.906	0.545	1.507	0.581
	> 30	0.851	0.498	1.455	0.821	0.692	0.378	1.267	0.312	0.959	0.573	1.605	0.959
Maternal marital status^d^	Married	0.844	0.699	1.019	0.077	0.845	0.696	1.025	0.088	0.919	0.768	1.100	0.356
Maternal education^e^	High school	1.088	0.847	1.398	0.269	0.998	0.767	1.299	0.468	1.060	0.832	1.349	0.096
	> High school	1.164	0.904	1.499	0.871	1.028	0.790	1.336	0.678	1.194	0.939	1.519	0.925
	College graduate	1.514	1.148	1.996	< 0.001	1.218	0.917	1.619	0.067	1.570	1.209	2.040	< 0.001
Poverty status^f^	Below poverty line	1.033	0.836	1.276	0.505	0.943	0.744	1.195	0.311	0.980	0.801	1.197	0.384
	Unknown	0.907	0.629	1.306	0.525	1.154	0.804	1.658	0.328	0.784	0.556	1.106	0.169
Number of vaccination providers for child^g^	2	0.842	0.699	1.013	0.950	0.784	0.648	0.950	0.285	0.743	0.624	0.885	0.398
	3+	0.699	0.506	0.965	0.089	0.782	0.568	1.076	0.432	0.657	0.488	0.883	0.066
Type of vaccination providers for child^h^	Other/mixed	1.307	0.979	1.745	0.327	1.240	0.923	1.666	0.041	1.438	1.101	1.876	0.016
	Private	1.407	1.069	1.852	0.023	1.003	0.759	1.325	0.271	1.327	1.026	1.717	0.243
Had Medicaid or SCHIP^i^	Yes	0.855	0.707	1.034	0.106	0.762	0.612	0.949	0.015	0.881	0.733	1.060	0.181

LCL = lower confidence limit; SCHIP = State Children's Health Insurance Program; UCL = upper confidence limit.

a All parameter estimates, odds ratios, and confidence intervals are weighted based on National Immunization Survey sampling design.

b Compared to children who did not have a combination vaccine (single-antigen only).

c Compared to children with mothers aged 19 years or younger.

d Compared to children with unmarried mothers.

e Compared to children with mothers with less than 12 years of schooling.

f Compared to children in households living above the poverty line.

g Compared to children with 1 vaccine provider.

h Compared to children with a public vaccine provider.

i Compared to children without Medicaid or SCHIP insurance.

## Discussion

The present study sought to examine the effect of combination vaccines on completion and compliance of childhood vaccination in the US using a nationally representative survey of households with children. Among children who received at least 1 classifiable vaccine by 24 months of age, we found 86% received at least 1 combination vaccine. The remaining 14% of the sample received single-antigen vaccines with no evidence of a combination vaccine. Children who received combination vaccines were demographically different than those who received single-antigen vaccine only. Specifically, children with single-antigen only vaccines had a significantly higher proportion of mothers who were married, graduated college, and were living above the poverty level. Prior studies have found that parents who refuse or intentionally delay vaccines tend to belong to households with higher income, have married mothers with college educations, and are covered by private health insurance.^12,5^ This suggests that children who received single-antigen vaccines are similar to those whose parents intentionally refuse or delay vaccinations.

Factors contributing to parental vaccine hesitancy (i.e., concern about whether to vaccinate one's child) have been widely examined. Evidence suggests vaccine refusal and delay may be attributed to perceived risks, perceived benefits, and vaccine safety or efficacy concerns.^4,5, 13-16^ As described by Salmon and colleagues,^17^ the impact of vaccine hesitancy is on a continuum. Vaccine hesitant parents may intentionally refuse vaccination, delay some or all vaccines (e.g., use of an alternative schedule), or accept vaccination for their child despite concerns. A 2012 survey of parents found that among those with children aged 2 to 6 y, 5.0% of parents delayed and 5.4% refused at least one recommended vaccine.^18^ Compared to the same survey conducted in 2014, researchers found the proportion of parents who delayed at least one vaccine remained constant (5.5%) while the proportion of those who refused at least one vaccine decreased significantly (3.3%, *P* < 0.05). Although the 2012 NIS does not include variables allowing direct measurement of vaccine refusal or delay, we anticipate a proportion of children who were undervaccinated or delayed vaccination could be attributed to vaccine hesitancy.

The use of combination vaccines is suggested as an effective strategy for increasing vaccination rates.^9^ In our study, we found completion rates were higher among children who received at least 1 combination vaccine. For 4:3:1:3:3:1:4 series, 69% of children receiving combination vaccines completed the recommended doses compared with only 50% of children receiving single-antigen vaccines only. A similar effect was observed in a retrospective study of administrative Medicaid claims that compared completion rates between children who received at least 1 dose of the DTaP-HepB-IPV combination vaccine and children who received no doses of combination vaccine.^10^ Results showed children in the combination cohort had a higher completion rate for the recommended vaccination series as well as higher rates of individual antigens (i.e., DTaP, HepB, and IPV) compared with children in the reference cohort.

Furthermore, findings from the current study revealed that children receiving at least 1 combination vaccine had better compliance rates compared with those who received single-antigen vaccines only. Low compliance among children who received single-antigen vaccines only may be in part attributed to fears of simultaneous vaccination. Prior evidence suggests that fears concerning simultaneous vaccination may lead to vaccine avoidance or delay.^4-6^ A recent systematic review found that parents raised concerns regarding pain and distress associated with simultaneous injections at a single visit, which may lead to vaccination avoidance or delays.^6^ Furthermore, several studies indicate that approximately 76% to 78% of parents who delayed their child's vaccinations had vaccine safety concerns linked to multiple injections.^4,5^

Adjusted logistic regression models demonstrated more than a 2-fold increase in the likelihood of being complete or compliant with the vaccine series by 24 months for children who received combination vaccines compared with those who received single- antigen vaccines only. These findings are consistent with those demonstrated in prior studies that compared compliance between children who received specific combination vaccines and single-antigen vaccines. Happe and colleagues (HAPPE 2007)^10^ reported that 45% of children who received DTaP-HepB-IPV received all the recommended vaccinations at age-appropriate times, whereas 38% of those who did not receive DTaP-HepB-IPV received their vaccinations on time. Additionally, a study by Marshall and colleagues^11^ found that any hepatitis B combination vaccine (DTaP-HepB-IPV or HepB-Hib) was associated with increased coverage rates for several individual vaccines and the full recommended childhood vaccination series.

Our study had several limitations, primarily related to restrictions of the NIS data set. Due to the use of telephone survey methods to identify household respondents and vaccination providers to collect information on vaccination history, estimates obtained from the NIS are susceptible to under-, inaccurate, or incomplete reporting. As completion and compliance measures rely on household- or provider-reported data, there is a possibility of bias in our measurements. Furthermore, the NIS lacks assessment of knowledge, attitudes, beliefs, healthcare seeking behavior, or other factors (e.g., reasons for vaccine refusal, availability/provider recommendation of single-antigen vaccines) related to vaccination that could provide a better understanding of reasons for administration of combination vaccines, receipt of single-antigen vaccines, and/or general vaccine hesitancy. As the 2 cohorts appear demographically different, future research should explore vaccine hesitancy as a potential confounder or moderator of combination vaccine uptake.

The present study fills a critical knowledge gap by evaluating the outcomes associated with combination vaccines. Prior studies that compared combination vaccines with single-antigen vaccines did not evaluate the effect of non–hepatitis B combination vaccines on compliance measures. Moreover, prior studies had not evaluated the effect of combination vaccines on completion and compliance of the full vaccination series recommended by the ACIP. Therefore, findings from the current study provide a more comprehensive understanding of the association between receipt of combination vaccines and completion or compliance outcomes. As vaccine compliance rates among children remain low in US, findings from this study suggest that combination vaccines should be encouraged for some children who are undervaccinated or are receiving single-antigen vaccines to increase compliance. Among the proportion of children with vaccine hesitant parents, particularly those with vaccine safety concerns, new strategies toward increasing vaccination are needed. Findings from the present study indicate differences in compliance between the combination and single-antigen cohorts for vaccines not included in a combination vaccine (e.g., rotavirus), suggesting issues other than simultaneous vaccination may impact vaccine delay/refusal. Interventions aimed at reducing vaccine hesitancy have been widely published; however, a recent systematic literature review found many interventions were not designed or powered to assess impact on vaccine refusal or delay.^19^ Therefore, future research aimed at evaluating the impact of strategies aimed at improving vaccination, including increasing the use of combination vaccines (e.g., increasing awareness of the benefits of the use of combination vaccines, educating parents and physicians who may be hesitant to use combination vaccines) is warranted.

## Patients and methods

### Data source

We used data from the 2012 National Immunization Survey (NIS) Public Use File.^20^ The NIS, conducted by the National Center for Health Statistics and the Centers for Disease Control and Prevention, has been used extensively for tracking vaccination rates among children in the US.^2,3^ The NIS consists of a nationally representative annual household survey of caregivers of young children to collect demographic and vaccination information as well as vaccination history from children's immunization providers. Demographic variables (e.g., race and ethnicity) were taken from the Household Survey as reported by the child's caregiver. Vaccination history (i.e., age in days at vaccination) was taken from the Provider Survey. Detailed NIS methods and Institutional Review Board (IRB) approval for data analysis are reported elsewhere.^21,22^ RTI International's IRB determined that this study met all criteria for exemption (RTI IRB ID Number: 13523).

### Study sample

From an unweighted sample of 25 736 children, we selected data for children who had complete survey responses from their caregivers, resided in the US (excluding the US Virgin Islands), and had adequate vaccination data supplied by their vaccine providers. Adequate vaccination data are a variable existing in the NIS denoting whether a child had missing or incomplete provider-reported vaccination data due to a lack of parental consent to contact the child's immunization provider(s), provider nonresponse, or incomplete provider-reported data. Excluding children with inadequate provider-reported vaccinations is a method used by the CDC when calculating annual vaccination completion using NIS data.^23^ Children aged less than 24 months at the time of the survey were excluded, thus capturing only children aged 24 to 35 months and allowing a consistent vaccination capture period between birth and 24 months for all children in the sample. Children who were unvaccinated or received only vaccines that could not be categorized as single antigen or combination (i.e., coded as “not otherwise specified”) were also excluded (N = 149). The final unweighted sample size was 11 561, equating to a weighted sample of 4 043 159 children.

### Study measures

#### Vaccine completion

Based on ACIP's 2012 recommendations,^24^ we defined vaccine completion as the accrual of the required number of doses by a specific age (8, 18, and 24 months) regardless of timing of vaccine administration. Detailed lists of doses required at 8, 18, and 24 months are presented elsewhere.^25^

Completion of rotavirus vaccine and *Haemophilus influenzae* type b vaccine (Hib) doses was assessed based on the product administered. For example, a child's vaccination for rotavirus was considered complete if 2 doses of *Rotarix* (GSK Vaccines) or 3 doses of *RotaTeq* (Merck & Co., Inc.) were administered. These algorithms are detailed in the published vaccine schedule.^24^

Although influenza and hepatitis A vaccines are also recommended by the ACIP, we did not assess them in the present study. The NIS data do not provide vaccination dates, thus we were not able to determine whether the seasonal influenza vaccine was administered during the influenza season, which is a criterion for administration. The second dose of hepatitis A vaccine should be administered between 6 and 18 months following the first dose at age 12 months or later; therefore, the second dose may be administered after 24 months—falling outside of our study period. As we could not capture appropriate completion and compliance of 2 doses of hepatitis A vaccine, it was not included in the present analysis.

#### Vaccine compliance

Vaccine compliance is a measure of the timeliness of vaccine administration. The ACIP suggests specific ages and intervals at which vaccines should be administered to maximize effectiveness.^24^ Vaccine compliance was measured as the proportion of children who received vaccine doses at age-appropriate intervals (e.g., dose 1 of DTaP at 2 months). Age-appropriate windows were based on the ACIP's recommended dosing schedule.^17,24^

Additionally, we measured compliance by calculating the number of days that a child was undervaccinated. This method has been proposed in several prior vaccine compliance studies.^2,3,26^ Undervaccination was assessed as having no evidence of a recommended vaccination 1 day following the end of the age-appropriate window. Each day of undervaccination was counted as 1 day, irrespective of the number of vaccines missed by that day.^27^ For a child who did not receive a vaccine by age 24 months, we calculated the total number of undervaccinated days as the number of days between the first day of undervaccination and age 24 months. The final measure of vaccine compliance was the total number of days undervaccinated. Accumulation of 7 months or more of undervaccination was categorized as “severely undervaccinated.” In addition to reporting total compliance with each dose, we also assessed the degree of compliance, including all, some, and no doses of recommended vaccine received on time.

The ACIP allows early vaccination as “age-appropriate” if received within a 4-day grace period before the minimum age for the dose.^9^ Any vaccines administered during a 4-day grace period before the minimum age for the dose were included as on time. Any vaccines administered before the grace period were excluded from calculation of the compliance end point.

#### Combination vaccines

We categorized all vaccines into 2 mutually exclusive groups: combination or single antigen. Table 5 lists vaccines that were considered combination vaccines. Vaccines with an unknown type (e.g., HI = “Hib, Unknown”) were not categorized as combination or single, but were included in calculating the completion and compliance endpoints. All other vaccines were considered to be single antigen.

**Table 5.** Vaccines considered to be combination vaccines.

CVX Code	Vaccine Components
07	DTaP-Hib
08	DTaP-HepB-IPV
D3	DTaP-IPV-Hib
43	HepB-Hib
VM	MMR-V

DTaP = diphtheria, tetanus toxoid, and acellular pertussis; HepB = hepatitis B; Hib = *Haemophilus influenzae* type b; IPV = inactivated poliovirus; MMR-V = measles, mumps, rubella, and varicella.

Although vaccines such as DTaP include multiple components in a single syringe, they were grouped with single-antigen vaccines. Preliminary review of the data indicated that no participants received the individual components of DTaP as single-antigen vaccines, and a small number of participants received a single-antigen measles vaccine; therefore, we anticipated that patterns of administration of DTaP and measles, mumps, and rubella (MMR) vaccines may be closer to those of true single-antigen vaccines (e.g., IPV) than to those of combination vaccines (e.g., DTaP-HepB-IPV).

After each vaccine was coded as combination or single antigen, the vaccination history of each child was reviewed to determine whether each child received at least 1 combination vaccine or single-antigen vaccines only. Next, a binary categorical measure of vaccine type was created to flag each child into 2 mutually exclusive groups: received at least 1 combination vaccine or received no combination vaccines (i.e., received single-antigen vaccines only). This categorical measure of vaccination type was used to determine differences in vaccination completion and compliance between children who received at least 1 combination vaccine and those who received single-antigen vaccines only.

### Statistical analyses

Bivariate analysis included evaluation of completion and compliance rates for the overall population and stratified by vaccination type. The Rao-Scott F-adjusted chi-square statistic was used to determine the differences between completion and compliance rates by vaccination type. This statistic is adjusted for complex survey data and provides a more conservative interpretation than the Wald chi-square statistic.^28^

Multivariable analysis included logistic regressions on 3 vaccination outcomes: completed vaccines series by 24 months (completion), received all vaccines on time (compliance), and undervaccinated for less than 7 months (compliance). These logistic regression models evaluated the effect of receipt of at least 1 combination vaccine on vaccine completion or compliance after adjusting for demographic characteristics such as maternal age, maternal marital status, maternal education, poverty status, enrollment in Medicaid or the State Children's Health Insurance Program (SCHIP), and provider variables including number of vaccination providers and type of vaccination providers (e.g., public, private).

Estimated frequencies, percentages, means, standard errors, medians, odds ratios, confidence intervals, and *P* values were calculated using SAS statistical software, version 9.3 (SAS Institute, Inc., 2011). A *P* value less than 0.05 was used as an indicator that a difference between groups may exist. SAS survey procedures and domain analysis techniques were used to calculate weighted values and correct standard errors per NIS data user guidance.^23^

## Trademark statements

*Rotarix* is a trademark of the GSK group of companies. *RotaTeq* is a trademark of Merck & Co., Inc.

## Prior presentation

Parts of this study were presented as abstract and oral presentation at the 18^th^ Annual Conference on Vaccine Research of the National Foundation for Infectious Diseases, Bethesda, MD, April 13–15, 2015.

## Note

The analyses, interpretations and conclusions presented here do not necessarily reflect the views or opinions of the National Center for Health Statistics, which is responsible only for the initial data.

## Abbreviations

ACIP: Advisory Committee on Immunization Practices
DTaP: diphtheria, tetanus toxoid, and acellular pertussis vaccine
HepB: hepatitis B vaccine
Hib: Haemophilus influenzae type b vaccine
IPV: inactivated poliovirus vaccine
IRB: institutional review board
LCL: lower confidence limit
MMR: measles, mumps, and rubella vaccine
MMR-V: measles, mumps, rubella, and varicella vaccine
NIS: National Immunization Survey
PCV: pneumococcal conjugate vaccine
SCHIP: State Children's Health Insurance Program
SE: standard error of the mean
UCL: upper confidence limit
US: United States

## References

[1] Robinson CL, Romero JR, Kempe A, Pellegrini C. Advisory Committee on Immunization Practices (ACIP) Child/Adolescent Immunization Work Group Advisory Committee on Immunization Practices Recommended Immunization Schedule for Children and Adolescents Aged 18 Years or Younger – United States, 2017. MMWR Morb Mortal Wkly Rep. 2017;66(5):134-135. doi:10.15585/mmwr.mm6605e1. PMID:28182607 28182607 PMC5657960

[2] Luman ET, McCauley MM, Stokley S, Chu SY, Pickering LK Timeliness of childhood immunizations. Pediatrics. 2002;110(5):935-9doi:10.1542/peds.110.5.935. PMID:12415033 12415033

[3] Luman ET, Barker LE, Shaw KM, McCauley MM, Buehler JW, Pickering LK Timeliness of childhood vaccinations in the US: days undervaccinated and number of vaccines delayed. JAMA. 2005;293(10):1204-11. doi:10.1001/jama.293.10.1204. PMID:15755943 15755943

[4] Gust DA, Darling N, Kennedy A, Schwartz B Parents with doubts about vaccines: which vaccines and reasons why. Pediatrics. 2008;122(4):718-25. doi:10.1542/peds.2007-0538. PMID:18829793 18829793

[5] Smith PJ, Humiston SG, Parnell T, Vannice KS, Salmon DA The association between intentional delay of vaccine administration and timeline childhood vaccination coverage. Public Health Rep. 2010;125(4):534-41. doi:10.1177/003335491012500408. PMID:20597453 20597453 PMC2882604

[6] Wallace AS, Mantel C, Mayers G, Mansoor O, Gindler JS, Hyde TB Experiences with provider and parental attitudes and practices regarding the administration of multiple injections during infant vaccination visits: lessons for vaccine introduction. Vaccine. 2014;32(41):5301-10. doi:10.1016/j.vaccine.2014.07.076. PMID:25092632 25092632

[7] Luthy KE, Beckstrand RL, Peterson NE Parental hesitation as a factor in delayed childhood immunization. J Pediatr Health Care. 2009;23(6):388-93. doi:10.1016/j.pedhc.2008.09.006. PMID:19875026 19875026

[8] Cohen NJ, Lauderdale DS, Shete PB, Seal JB, Daum RS Physician knowledge of catch-up regimens and contraindications for childhood immunizations. Pediatrics. 2003;111(5):925-32. doi:10.1542/peds.111.5.925. PMID:12728067 12728067

[9] Centers for Disease Control and Prevention General recommendations on immunization. MMWR Morb Mortal Wkly Rep. 2011;60(2):1-60.

[10] Happe LE, Lunacsek OE, Marshall GS, Lewis T, Spencer S Combination vaccine use and vaccination quality in a managed care population. Am J Manag Care. 2007;13(9):506-12. PMID:17803364 17803364

[11] Marshall GS, Happe LE, Lunacsek OE, Szymanski MD, Woods CR, Zahn M, Russell A Use of combination vaccines is associated with improved coverage rates. Pediatr Infect Dis J. 2007;26(6):496-500. doi:10.1097/INF.0b013e31805d7f17. PMID:17529866 17529866

[12] Smith PJ, Chu SY, Barker LE Children who have received no vaccines: who are they and where do they live? Pediatrics. 2004;114(1):187-95. doi:10.1542/peds.114.1.187. PMID:15231927 15231927

[13] Gowda C, Dempsey AF The rise (and fall?) of parental vaccine hesitancy. Hum Vaccin Immunother. 2013;9(8):1755-62. doi:10.4161/hv.25085. PMID:23744504 23744504 PMC3906278

[14] Nadeau JA, Bednarczyk RA, Masawi MR, Meldrum MD, Santilli L, Zansky SM, Blog DS, Birkhead GS, McNutt LA Vaccinating my way–use of alternative vaccination schedules in New York State. J Pediatr. 2015;166(1):151-6. doi:10.1016/j.jpeds.2014.09.013. PMID:25444525 25444525

[15] Smith PJ, Humiston SG, Marcuse EK, Zhao Z, Dorell CG, Howes C, Hibbs B Parental delay or refusal of vaccine doses, childhood vaccination coverage at 24 months of age, and the Health Belief Model. Public Health Rep. 2011;126(Suppl 2):135-46. doi:10.1177/00333549111260S215. PMID:21812176 21812176 PMC3113438

[16] McCauley MM, Kennedy A, Basket M, Sheedy K Exploring the choice to refuse or delay vaccines: a national survey of parents of 6- through 23-month-olds. Acad Pediatr. 2012;12(5):375-83. doi:10.1016/j.acap.2012.06.007. PMID:22921495 22921495

[17] Salmon DA, Dudley MZ, Glanz JM, Omer SB Vaccine hesitancy. Causes, consequences, and a call to action. Vaccine. 2015;33:D66-71. doi:10.1016/j.vaccine.2015.09.035. PMID:26615171 26615171

[18] Frew PM, Fisher AK, Basket MM, Chung Y, Schamel J, Weiner JL, Mullen J, Omer SB, Orenstein W Changes in childhood immunization decisions in the United States: Results from 2012 & 2014 National Parental Surveys. Vaccine. 2016;34(46):5689-5696. doi:10.1016/j.vaccine.2016.08.001. PMID:27720447 27720447 PMC6026855

[19] Jarrett C, Wilson R, O'Leary M, Eckersberger E, Larson HJ. SAGE Working Group on Vaccine Hesitancy Strategies for addressing vaccine hesitancy – A systematic review. Vaccine. 2015;33(34):4180-90. doi:10.1016/j.vaccine.2015.04.040. PMID:25896377 25896377

[20] U.S. Department of Health and Human Services (DHHS). National Center for Health Statistics. The 2012 National Immunization Survey. Hyattsville, MD: Centers for Disease Control and Prevention; 2013.http://www.cdc.gov/nchs/nis/data_files.htm

[21] Smith PJ, Battaglia MP, Huggins VJ, Hoaglin DC, Rodén A, Khare M, Ezzati-Rice TM, Wright R Overview of the sampling design and statistical methods used in the National Immunization Survey. Am J Prev Med. 2001;20(4 Suppl):17-24. doi:10.1016/S0749-3797(01)00285-9. PMID:11331127 11331127

[22] Zell E, Ezzati-Rice TM, Battaglia M, Wright R National Immunization Survey: the methodology of a vaccination surveillance system. Public Health Rep. 2000;115(1):65-77. doi:10.1093/phr/115.1.65. PMID:10968587 10968587 PMC1308558

[23] Centers for Disease Control and Prevention (CDC), National Center for Immunization and Respiratory Diseases (NCIRD), National Center for Health Statistics. National Immunization Survey. A user's guide for the 2012 public-use data file NORC at the University of Chicago; 2014.

[24] Centers for Disease Control and Prevention (CDC). Recommended immunization schedules for persons aged 0 through 18 years—United States, 2012. MMWR Morb Mortal Wkly Rep. 2012;61(5):1-4. 22451974

[25] Kurosky SK, Davis KL, Krishnarajah G Completion and compliance of childhood vaccinations in the United States. Vaccine. 2016;34(3):387-94. doi:10.1016/j.vaccine.2015.11.011. PMID:26597149 26597149

[26] Glanz JM, Newcomer SR, Narwaney KJ, Hambidge SJ, Daley MF, Wagner NM, McClure DL, Xu S, Rowhani-Rahbar A, Lee GM, et al. A population-based cohort study of undervaccination in 8 managed care organizations across the United States. JAMA Pediatr. 2013;167(3):274-81. doi:10.1001/jamapediatrics.2013.502. PMID:23338829 23338829

[27] Luman ET, Barker LE, McCauley MM, Drews-Botsch C Timeliness of childhood immunizations: a state-specific analysis. Am J Public Health. 2005;95(8):1367-74. doi:10.2105/AJPH.2004.046284. PMID:16043668 16043668 PMC1449368

[28] Rao JNK, Scott A “The Analysis of Categorical data from complex sample surveys: chi-squared tests for goodness of fit and independence in two-way tables,” J Am Statistical Association. 1981;76:221-230. doi:10.1080/01621459.1981.10477633

